# The SLC25 Mitochondrial Carrier Family: Structure and Mechanism

**DOI:** 10.1016/j.tibs.2019.11.001

**Published:** 2019-11-29

**Authors:** Jonathan J. Ruprecht, Edmund R.S. Kunji

**Affiliations:** 1 Medical Research Council (MRC) Mitochondrial Biology Unit, University of Cambridge, Cambridge Biomedical Campus, Cambridge, CB2 0XY, UK

## Abstract

Members of the mitochondrial carrier family (SLC25) provide the transport steps for amino acids, carboxylic acids, fatty acids, cofactors, inorganic ions, and nucleotides across the mitochondrial inner membrane and are crucial for many cellular processes. Here, we use new insights into the transport mechanism of the mitochondrial ADP/ATP carrier to examine the structure and function of other mitochondrial carriers. They all have a single substrate-binding site and two gates, which are present on either side of the membrane and involve salt-bridge networks. Transport is likely to occur by a common mechanism, in which the coordinated movement of six structural elements leads to the alternating opening and closing of the matrix or cytoplasmic side of the carriers.

## The Mitochondrial Carrier Family

**Figure 1:**
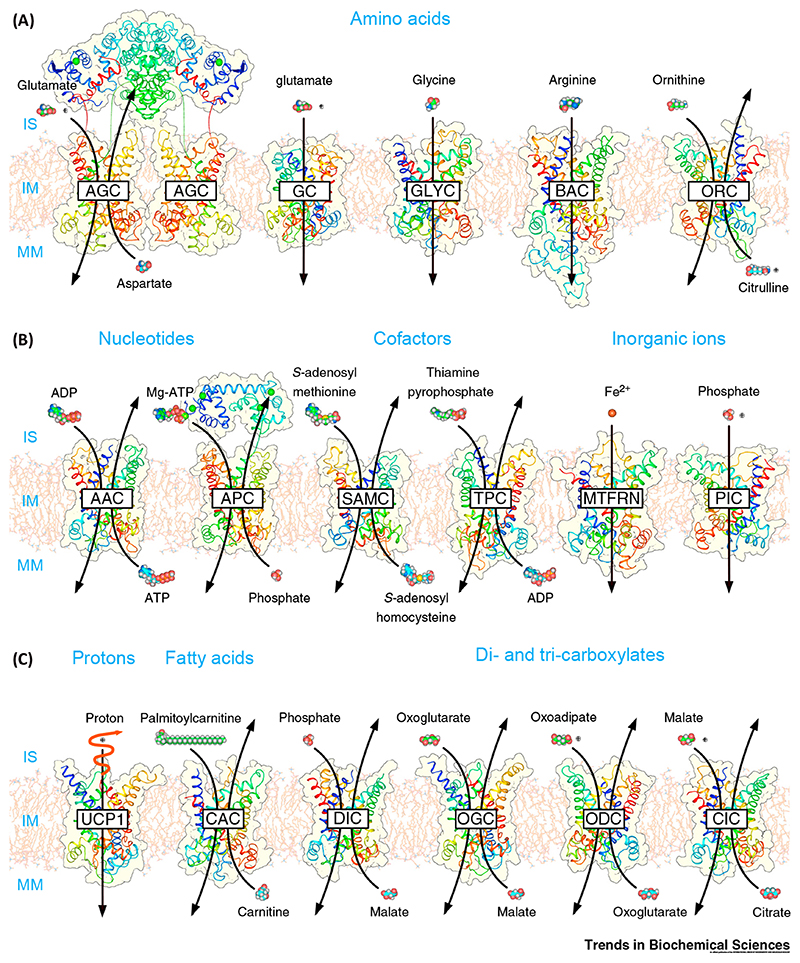
Selected Transporters of the Human Mitochondrial Carrier Family (SLC25). Mitochondrial carriers transport (A) amino acids; (B), nucleotides, cofactors, and inorganic ions; and (C) protons, fatty acids, and di- and tricarboxylates across the mitochondrial inner membrane using antiport, symport, and uniport activities [78]. The ATP-Mg/Pi carrier and aspartate glutamate carriers have a three-domain structure, comprising a calcium-regulated domain, a carrier domain, and a domain with an amphipathic helix. Protons are shown as grey spheres with a plus sign. The structures are homology models based upon PDB: 1OKC and 4C9H chain A, and generated by Swiss-Model [79]. The regulatory domains of AGC and APC are based upon PDB: 4P5W and 4ZCU, respectively, and the links to the carrier domains are not modelled. Abbreviations: AAC, ADP/ATP carrier (SLC25A4, SLC25A5, SLC25A6, SLC25A31); AGC, aspartate glutamate carrier (SLC25A12, SLC25A13); APC, ATP-Mg/Pi carrier (SLC25A23, SLC25A24, SLC25A25); BAC, basic amino acid carrier (SLC25A29); CAC, carnitine-acylcarnitine carrier (SLC25A20); CIC, citrate (tricarboxylate) carrier (SLC25A1); DIC, dicarboxylate carrier (SLC25A10); GC, glutamate carrier (SLC25A18, SLC25A22); GLYC: glycine carrier (SLC25A38); IM inner membrane; IS, intermembrane space; MM mitochondrial matrix; MTFRN mitoferrin (SLC25A28, SLC25A37); ODC, oxoadipate carrier (SLC25A21); OGC, oxoglutarate carrier (SLC25A11); ORC, ornithine carrier (SLC25A2, SLC25A15); PIC, phosphate carrier (SLC25A3); SAMC, S-adenosylmethionine carrier (SLC25A26); TPC, thiamine pyrophosphate carrier (SLC25A19); UCP1, uncoupling protein (SLC25A7).

With 53 members, the mitochondrial carrier family (solute carrier family 25, SLC25) is the largest solute transporter family in humans. They transport solutes across the impermeable inner membrane of mitochondria for important cellular processes, such as oxidative phosphorylation of fats and sugars, amino acid catabolism and interconversion, synthesis of iron sulfur clusters and heme, macromolecular synthesis, and heat production (Figure 1). Approximately one-third of human mitochondrial carriers are currently orphan transporters, with no known substrate. Most operate as strict counter-exchangers of chemically related substrates [**antiporters** (see Glossary)], but some display unidirectional (**uniporters**) or substrate–proton (**symporters**) transport activities. In this review, we focus on the role and properties of some of the best characterized members of the family, which will be introduced first.

Nucleotide transporters include the mitochondrial ADP/ATP carrier, also called adenine nucleotide translocase or translocator (ANT), which imports ADP into the mitochondrial matrix, where it can be converted to ATP by ATP synthase, and exports the newly synthesized ATP to the cytosol, where it fuels the metabolic energy-requiring processes that are vital for cell survival [1–5]. There are four different isoforms in humans, AAC1, AAC2, AAC3, and AAC4 (SLC25A4, SLC25A5, SLC25A6, and SLC25A31, respectively), which are expressed in a tissue-dependent manner [6]. Mitochondrial ATP-Mg/Pi carriers carry out the electroneutral antiport of ATP-Mg (but also ATP, ADP, and AMP) and Pi, and can therefore change the mitochondrial adenine nucleotide pool [7–9]. Atypically, they consist of three domains: an N-terminal calcium-regulatory domain with four **EF-hands**, an amphipathic helix, and a C-terminal carrier domain, which transports substrates [10–12]. In the presence of calcium, the amphipathic helix binds to the regulatory domain [11,13], whereas in its absence it binds to the carrier domain, inhibiting transport [10,12]. There are three human isoforms, APC1 (SLC25A24), APC2 (SLC25A23), and APC3 (SLC25A25) [8], which have the three-domain structure [11], and a fourth isoform APC4 (SLC25A41), which lacks the regulatory domain [14].

Inorganic ion transporters include the mitochondrial phosphate carrier PIC (SLC25A3) and the uncoupling protein UCP1 (SLC25A7). PIC imports phosphate in symport with a proton for the synthesis of ATP [15–17]. UCP1 is found predominantly in brown adipose tissue of neonatal mammals and dissipates the proton motive force, which is converted to heat [18–20]. It is activated by fatty acids and inhibited by purine nucleotides [21], but the mechanism is still debated [22–26].

The aspartate/glutamate carriers AGC1 (SCL25A12) and AGC2 (SLC25A13) are examples of amino acid transporters. They import glutamate in symport with a proton and export aspartate, and they function in the malate-aspartate shuttle, gluconeogenesis, the urea cycle (AGC2-specific), and myelin synthesis (AGC1-specific) [27,28]. They are calcium regulated and have an unusual three-domain structure consisting of an N-terminal calcium-regulatory domain containing eight EF-hands, a carrier domain, and a C-terminal amphipathic helix [29]. Unexpectedly, the regulatory domain forms a dimerization interface [29], which is a unique feature among mitochondrial carriers, which are otherwise monomeric [30].

Other physiologically important family members include the thiamine pyrophosphate transporter TPC (SLC25A19) [31], the carnitine/acylcarnitine carrier CAC (SLC25A20) [32–34], the mitochondrial oxoglutarate carrier OGC (SLC25A11) [35–37], and the tricarboxylate or citrate carrier CIC (SLC25A1) [38–41], substrates of which are shown in Figure 1.

In this review, we will describe the latest insights into the transport mechanism of the mitochondrial ADP/ATP carrier and investigate whether it is shared with other mitochondrial carriers by examining their structural and sequence properties.

## Structures of Mitochondrial ADP/ATP Carriers in Cytoplasmic- and Matrix-States

Most of the structural information for the SLC25 family comes from studies of the mitochondrial ADP/ATP carrier; this is due to the high natural abundance of this protein and the availability of inhibitors that trap the carrier in specific conformations. Atractyloside (ATR) and the chemically related carbox-yatractyloside (CATR) lock the carrier in a **cytoplasmic state (c-state)** in which the substrate-binding site is accessible to the intermembrane space, which is confluent with the cytosol [42–44]. Bongkrekic acid (BKA) and its isomer isobongkrekic acid lock the carrier in a **matrix state (m-state)** with the substrate-binding site accessible to the matrix [45,46]. The first structural information was obtained by electron crystallography of 2D crystals of the *Saccharomyces cerevisiae* ADP/ATP carrier ScAac3p, trapped in the c-state by ATR [47]. The projection maps showed that the carrier was monomeric with threefold pseudo-symmetry and had a central substrate translocation pathway. The first atomic structure of the bovine ADP/ATP carrier inhibited by CATR was determined by X-ray crystallography (PDB ID: 1OKC and 2C3E) [48]. The structure revealed a barrel-shaped protein composed of three domains related by threefold pseudo-symmetry. Each domain is composed of an odd-numbered transmembrane α-helix (H1, H3, or H5), a loop containing a short matrix α-helix (h12, h34, or h56) that lies parallel to the membrane plane, and an even-numbered transmembrane α-helix (H2, H4, or H6).

**Figure 2:**
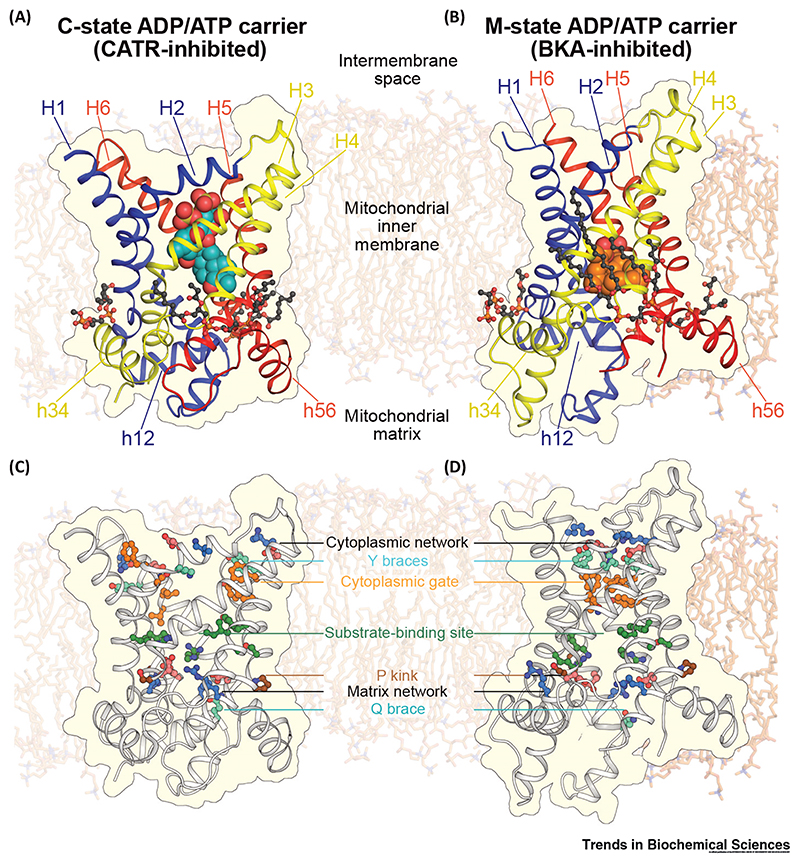
Key Structural and Functional Elements of Mitochondrial Carriers. Structures of (A) the cytoplasmic state (c-state) of the mitochondrial ADP/ATP carrier trapped by the inhibitor carboxyatractyloside (CATR) (ScAac2p, PDB: 4C9H chain A [49]); and (B) the matrix state (m-state) ADP/ATP carrier trapped by the inhibitor bongkrekic acid (BKA) (TtAac, PDB: 6GCI chain A [54]). The proteins are shown in cartoon representation (blue, domain 1; yellow, domain 2; red, domain 3). Transmembrane α-helices (H1–H6) and matrix α-helices (h12, h34, and h56) are indicated. The inhibitors are shown as space-filling models, with blue-green carbon atoms for CATR and orange carbon atoms for BKA. The cardiolipin molecules are shown in ball-and-stick representation with dark grey, red, and orange balls for carbon, oxygen, and phosphorus atoms, respectively. Key functional elements, highlighted in different colors, in the (C) c- and (D) m-states, viewed in the same orientation as (A) and (B), respectively.

This structural fold was subsequently confirmed by structures of the yeast ADP/ATP carrier isoforms ScAac2p (PDB ID: 4C9G and 4C9H) and ScAac3p (PDB ID: 4C9J and 4C9Q), also trapped in the c-state by CATR (Figure 2A, C) [49]. The odd-numbered transmembrane α-helices have pronounced kinks, located at the proline residues of the highly conserved **signature motif** Px[DE]xx[KR] (P kink in Figure 2), giving them a pronounced L-shape, which helps to block access to the central cavity from the mitochondrial matrix in the c-state. Over 40% of ADP/ATP carrier sequences, including both ScAac2p and ScAac3p, have a serine substitution for the proline of the signature motif on H3, which forms a hydrogen bond to its own backbone amide group, mimicking the disruption proline causes to α-helical backbone hydrogen bonding [49]. The charged residues of the signature motifs form interdomain salt-bridges [48–50], now called the **matrix salt-bridge network** [51] (Figure 2), as predicted earlier by genetic analysis [52]. Residues of the salt-bridge between domains 1 and 3 interact with a proximal glutamine residue (Px[DE]xx[KR]xxxQ), which hydrogen bonds to both salt-bridge residues, forming a brace that stabilizes the matrix network (**Q brace** in Figure 2) [49]. The glutamine residues are highly conserved, and one to three Q braces are typically found in SLC25 members, bracing the salt bridges of the matrix network. CATR inhibits the ADP/ATP carrier by binding tightly in the central cavity, forming multiple salt-bridges and hydrogen bonds with protein residues, blocking the translocation pathway (Figure 2A) [48,49]. Three cardiolipin molecules are tightly bound to the carrier, bridging the matrix helices and the even-numbered transmembrane helices (Figure 2) [49,50]. The cardiolipin molecules play an important role in stabilizing their proximal interdomain interfaces and are tightly bound to the protein [53] by hydrogen bonds [48] and by electrostatic interactions with helix dipoles [49].

Recently, the first structure of a mitochondrial carrier in the m-state has been solved: the ADP/ATP carrier from the thermotolerant fungus *Thermothelomyces thermophila* (TtAac), inhibited by BKA (PDB ID: 6GCI; Figure 2B, D) [54]. The m-state structure shows the characteristic three-domain architecture, but with the domains rotated compared with the c-state, opening up the central cavity to the mitochondrial matrix and closing it to the intermembrane space. As a consequence, the matrix network and Q brace are disrupted (Figure 2D). On the intermembrane side, the transmembrane helices are positioned close together, allowing the charged residues of the [YF][DE]xx[KR] motifs on the even-numbered α-helices to form the interdomain **cytoplasmic salt-bridge network** (Figure 2D) [51,54]. This network is stabilized by hydrogen bonds with the hydroxyl groups of the tyrosines of the motif, forming a tyrosine brace (**Y brace** in Figure 2). Most SLC25 family members have one to three Y braces.

Comparison of the available c- and m-state structures indicates that both CATR and BKA induce subtle perturbations in the protein structure, which likely contribute to their inhibition mechanism [54]. Nevertheless, the structural information now available for both states has significantly advanced our understanding of how these proteins operate at the molecular level [5,54]. The structural features seen in the ADP/ATP carriers are likely to be conserved throughout the mitochondrial carrier family, with few exceptions.

## A Single Substrate-Binding Site

SLC25 proteins can transport substrates with exquisite specificity, as exemplified by the ADP/ATP carrier which transports only ADP or ATP or their deoxy variants, but not any other adenine or guanine nucleotides [55–57]. To understand how they achieve this, it is essential to know where substrates bind and how they interact with the protein. Unfortunately, the structures of the ADP/ATP carriers were solved using inhibitors that are chemically distinct from substrates and thus were unable to reveal the substrate-binding site directly.

**Figure 3:**
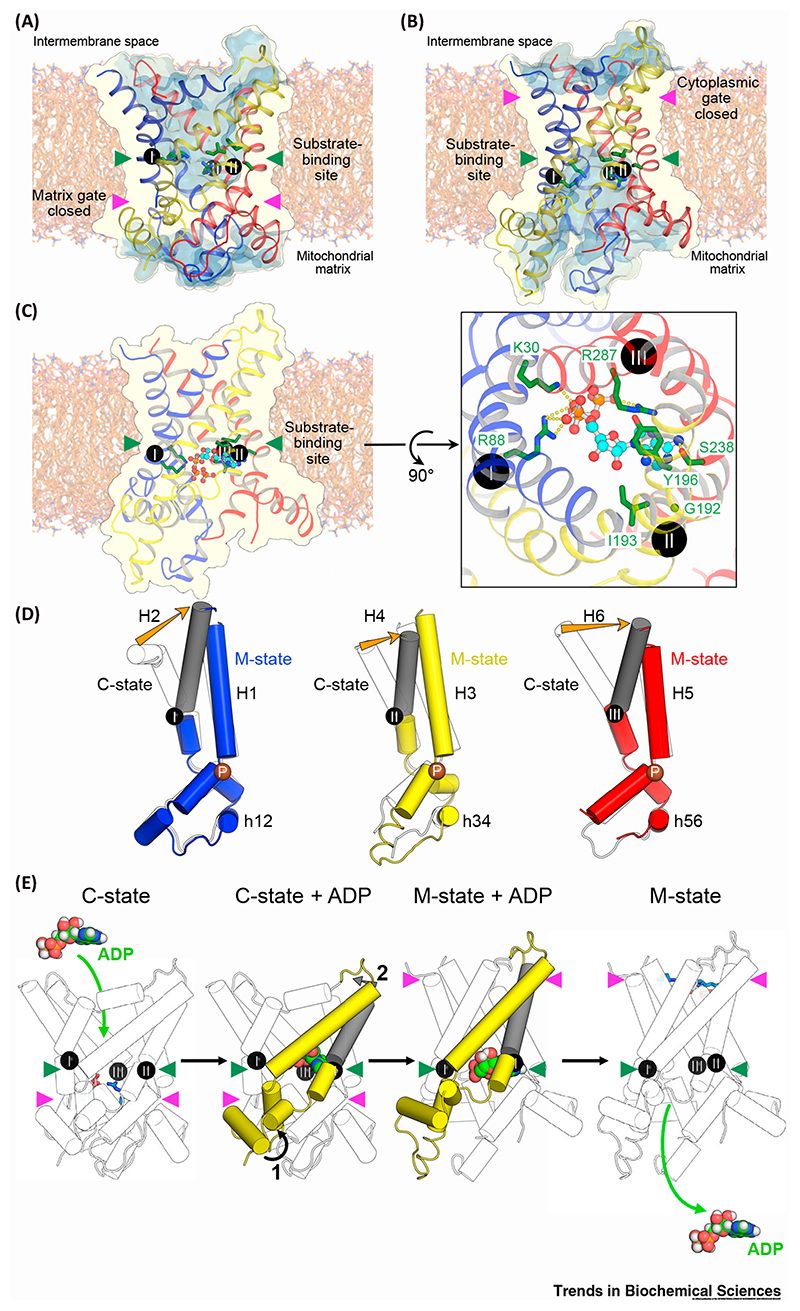
Substrate-Binding and Conformational Changes between Cytoplasmic- and Matrix-States. (A) The c-state of the mitochondrial ADP/ATP carrier (ScAac2p, PDB: 4C9H chain A), showing the substrate-binding site residues (green sticks) and matrix gate, and with the water-accessible surface in pale blue. (B) The m-state of the mitochondrial ADP/ATP carrier (TtAac, PDB: 6GCI chain A), showing the substrate-binding site residues (green sticks) and cytoplasmic gate, with the water-accessible surface in pale blue. (C) ATP docked into the proposed substrate-binding site of the m-state. Interactions between ATP and substrate-binding site residues are indicated by yellow dashed lines in the right-hand panel. (D) Comparison of the domain structures of the c-state, shown in outline, and m-state, shown with domains 1–3 colored blue, yellow, and red, respectively. The prolines of the conserved Px[DE]xx[KR] signature motifs are indicated by brown spheres. Orange arrows indicate the inward movement of the gate elements (shown in grey). (E) Conformational changes between c- and m-states, viewed laterally from the membrane. Matrix and cytoplasmic network residues are shown as sticks in the left- and right-hand panels, respectively. Conformational changes induced by substrate-binding involve (1) rotations of the core elements of each domain (highlighted here for domain 2), shown by a black arrow, and (2) inward movements of the gate elements (grey arrow). In (A–C) and (E), the position of the substrate-binding site is highlighted by green arrowheads and the gates are highlighted by magenta arrowheads. In (A–E), the contact points of the substrate-binding site are indicated by black spheres with Roman numerals.

Diverse bioinformatic and modeling approaches have been undertaken to try to pin-point the substrate-binding site. In one approach, homology models were probed with chemical and distance constraints to identify conserved residues that were capable of discriminating between keto acid and amino acid substrates, and adenine nucleotides [58,59]. A common substrate-binding site in the central cavity was identified, which includes residues on each of the even-numbered transmembrane helices, called the **contact points** (shown as black spheres with Roman numerals in Figure 3) [58,59]. Importantly, this analysis indicated that contact point II (on H4) plays the key role of discriminating between different classes of substrate (e.g., keto or amino acids), contact point I (on H2) can discriminate between different substrates within the same class, and contact point III (on H6) is nearly always a positively charged residue and therefore does not confer specificity [58,59]. A second approach was based upon the striking difference between the threefold pseudo-symmetry of the carriers and the asymmetric nature of the substrates they transport [51]. Carriers must have evolved asymmetric substrate-binding site residues to match their asymmetric substrates. From sequence information alone, a score was devised to reflect both the conservation and degree of symmetry of all residues in mitochondrial carriers [51]. For all mitochondrial carriers analyzed, a single cluster of asymmetric residues was identified, located in the middle of the membrane and overlapping with the substrate-binding site proposed earlier [58,59]. A third approach has used molecular dynamics to investigate the binding of ADP and ATP to residues in the c-state bovine ADP/ATP carrier structure, showing that electrostatic attraction was a major driving force for binding [56,60,61].

A consensus set of residues for substrate-binding can be identified from these studies, corresponding approximately to the middle of the membrane in both the c- (Figure 3A) and m-state structures (Figure 3B). They lie at the bottom of a water accessible cavity open to either the intermembrane space or mitochondrial matrix, respectively [5,48,49]. These features are consistent with an **alternating access** transport mechanism, which is common to most transport proteins [62,63]. For the ADP/ATP carrier, substrate-binding residues include the positively charged K30, R88, and R287 (TtAac numbering), which interact with the negatively charged phosphate groups of the substrate; G192, I193, and Y196, which form hydrophobic interactions with the adenine ring; and S238, which may form a hydrogen bond to the adenine ring (Figure 3C). R88 is part of contact point I (which discriminates between different substrates of the same class), G192, I193, and Y196 are part of contact point II (which distinguishes between different classes of substrate), and R287 is part of contact point III. Consistent with this notion, residue I183 of bovine AAC1, equivalent to I193, is crosslinked by azido-ADP [64]. Both CATR and BKA form polar interactions with the substrate-binding site residues and thus target a functionally critical part of the protein [48,49,54]. Since ADP has to be imported against the negative inside membrane potential, the carrier neutralizes ADP with the three formal positive charges of K30, R88, and R287 in the substrate-binding site [58,59]. In the export step, a net negative charge would remain for ATP, which will favor export. Biophysical measurements show that a net charge of+0.3 and –0.7 are moved across the membrane by transport of ADP and ATP, respectively, indicating that there are 3.3 positive counter-charges at the binding site [65,66], which agrees well.

The three contact points in the central substrate-binding site are universally conserved across the SLC25 family, indicating that the substrate-induced conformational changes will be similar [51,59]. Another common feature of substrate binding is neutralization of charge on the substrate, which is a requirement as most substrates for carriers are anions. Furthermore, carriers that transport substrates in symport with protons have negatively charged residues in their predicted substrate-binding sites, which simultaneously bind protons and substrate for coupled transport [17].

## Matrix and Cytoplasmic Salt-Bridge Networks and Gates

**Figure 4:**
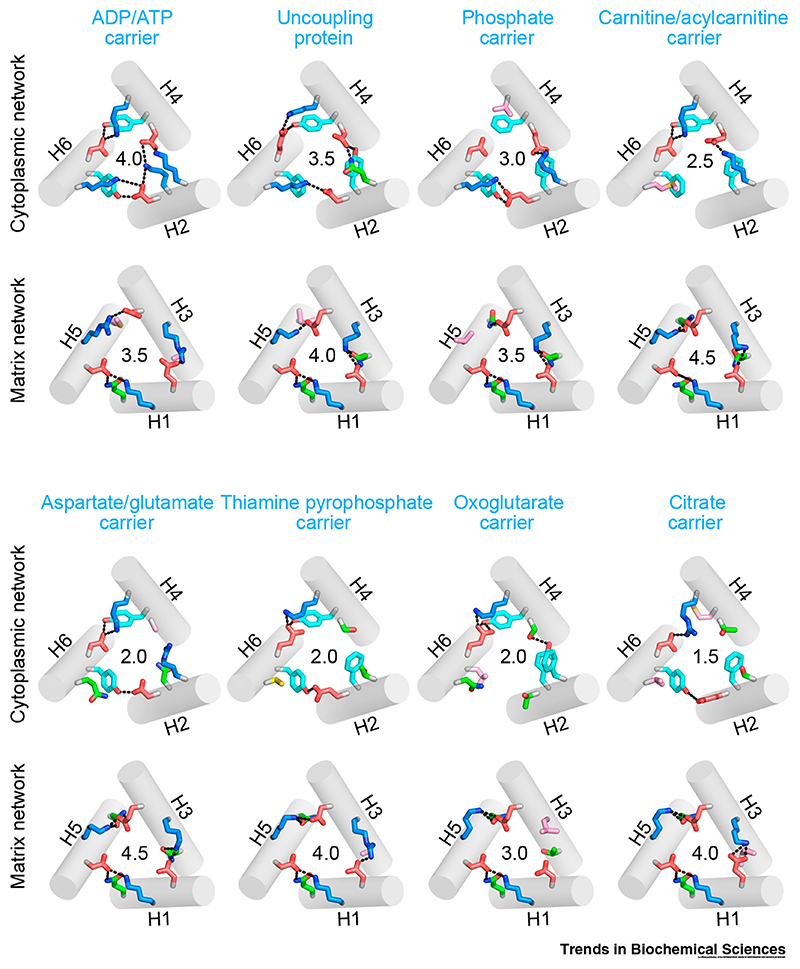
Relative Cytoplasmic and Matrix Network Strengths of Different SLC25 Family Members. Positively charged, negatively charged, polar, aliphatic, and aromatic residues are shown in blue, red, green, pink, and cyan colors, respectively. The bonds are shown as black dashes. The number in the center is the total interaction energy of the network, where salt-bridge interactions and braces are counted as 1.0 and 0.5, respectively. The models of the cytoplasmic network are derived from PDB:6GCI (chain A), whereas those of the matrix network are derived from PDB:1OKC.

In an alternating access mechanism, two **gates** open and close the transporter, exposing the substrate-binding site to one or other side of the membrane [62,63]. In mitochondrial carriers, the matrix salt-bridge network and glutamine brace (Px[DE]xx[KR]xxxQ) and the cytoplasmic salt-bridge network and tyrosine brace ([YF][DE]xx[KR]) are key components of the gates. Since these networks need to break and form during the transport cycle alternately, their interaction energies are likely to be important for the overall energetics of transport. The matrix salt-bridge network is highly conserved across the SLC25 family with relatively few substitutions [58,67], whereas the cytoplasmic salt-bridge network is more variable [51,68]. The number of glutamine braces [49] and tyrosine braces [54] differ between carriers, providing further modulation of the network interaction energies. Interestingly, when one residue of the bond is mutated, abolishing an ionic interaction, the interacting residue is often also mutated (Figure 4).

Our current understanding of the structure of the networks in different SLC25 members is based upon homology models of the m- and c-state. Since the precise geometry and distances of the interactions and the pH and ionic strength will affect the bonding strength, it is difficult to calculate an accurate interaction energy for the networks. However, salt-bridges involve interactions between formal charges at relatively short distances (2.8–3.0 Å), whereas hydrogen bonds involve interactions between partial charges at longer distances (3.0–3.5 Å). Consequently, hydrogen bonds are generally much weaker (1.5–7 kcal/mol) than salt-bridges (3–10 kcal/mol) [69]. Therefore, we introduced a semi-quantitative measure where the interaction energy of hydrogen bonds is taken to be half that of salt-bridges [49,51,70,71]. In this way, we can assign a comparative estimate of the interaction energy of each network for any given carrier (Figure 4).

In addition to the networks, other residues are involved in the formation of the matrix and cytoplasmic gate. The C-terminal ends of the odd-numbered helices are part of the gate in the c-state. The aromatic residues of the [YF][DE]xx[KR] motif, together with additional aromatic or hydrophobic residues with bulky side-chains, are part of the cytoplasmic gate in the m-state [5]. The gates are both approximately 15-Å thick, providing insulation against proton leak, which would dissipate the proton motive force, a key feature of energy conversion in mitochondria. The salt-bridge networks and gate residues are conserved across the SLC25 family, in agreement with their key role in the proposed mechanism.

## Conformational Changes in the Transport Cycle

Comparison of the domain structures of the CATR- and BKA-inhibited ADP/ATP carriers indicates that about 80% of the domain structure is conserved between the two states (Figure 3D) [54]. Conformational changes between states must, therefore, involve rigid-body motions of these core elements, which include the odd-numbered helices, the matrix helices, and one-third of the even-numbered helices. There is, however, a pronounced difference in the position of the C-terminal ends of the even-numbered helices in each domain, called the gate elements (highlighted in grey for the m-state domains in Figure 3D) [54]. A plausible transport mechanism for mitochondrial carriers, therefore, involves the core elements of each domain rocking outward during the transition from the c- to m-state, disrupting the matrix network and opening the substrate-binding site to the matrix side of the membrane (Figure 3E) [54]. Simultaneously, the gate elements rotate inwards, closing access to the substrate-binding site from the intermembrane space and allowing the cytoplasmic network to form (Figure 3E). Transition between the m- and c-states involves the same elements operating in reverse. Whilst it is unclear how substrate binding is coupled to these conformational changes, it appears significant that the contact points of the binding site are the hinges between the core and gate elements. The involvement of all three contact points in substrate binding is likely to play an important role in ensuring that the conformational changes occur concurrently in all three domains [5,54].

## Conserved Residues at Interhelical Interfaces

**Figure 5:**
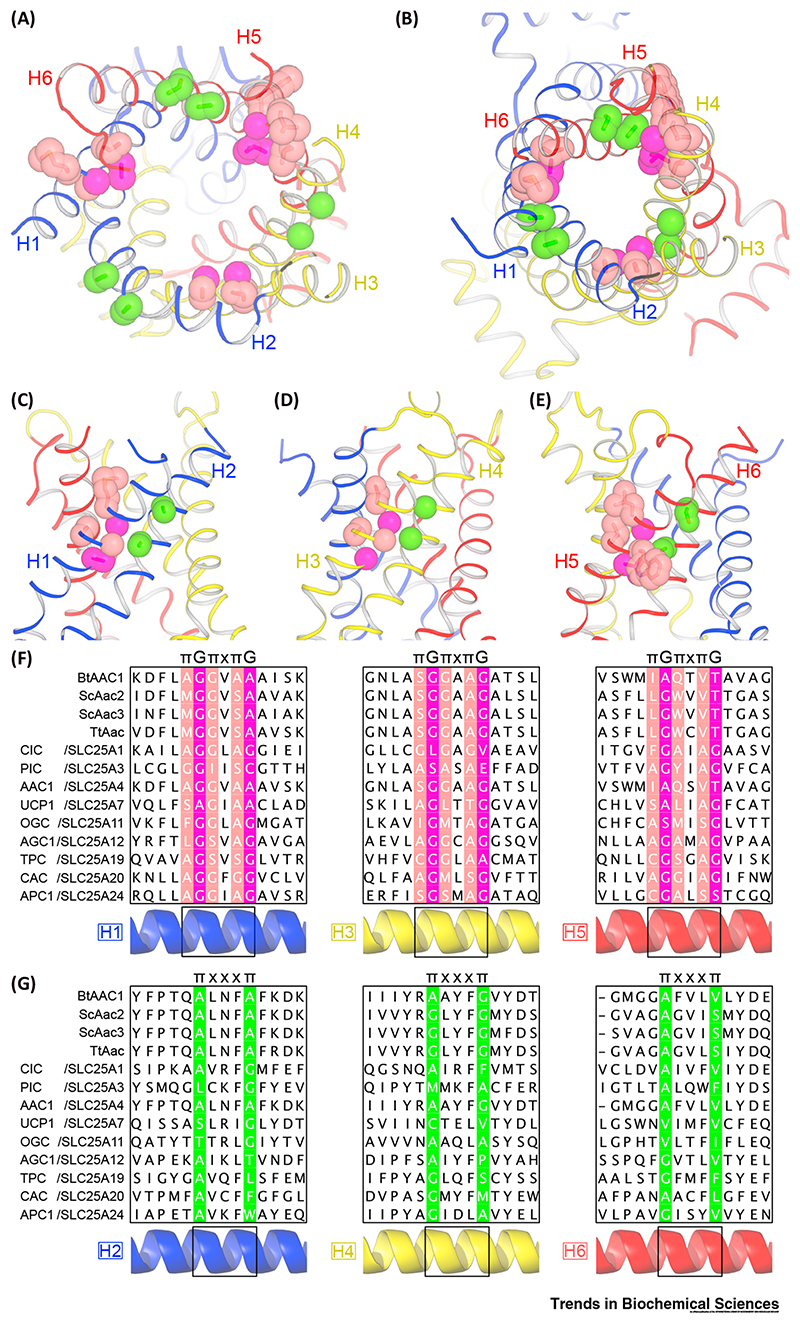
Conserved πGπxπG and πxxxπ Motifs Allow Close-Packing of Helices in the Matrix State (m-State). (A) Structure of the cytoplasmic state (c-state) of the mitochondrial ADP/ATP carrier (ScAac2p, PDB: 4C9H chain A); and (B) structure of the m-state (TtAac, PDB: 6GCI chain A), both viewed from the cytoplasmic side of the membrane (blue, domain 1; yellow, domain 2; red, domain 3). The motifs are shown in stick representation and with semitransparent spheres at the van der Waals radius of the appropriate atom. The πGπxπG motif is colored salmon and magenta at the positions of π and G residues, respectively. The πxxxπ motif is colored green at the position of the π residues. (C-E) Side views of the motifs at domain interfaces in the m-state, highlighting domain 1 (C), domain 2 (D), and domain 3 (E). (F) Amino acid sequences of selected mitochondrial carriers around the πGπxπG motif on H1 (left), H3 (middle), and H5 (right). (G) Amino acid sequences of selected mitochondrial carriers around the πxxxπ motif on H2 (left), H4 (middle), and H6 (right). In (F) and (G), residues of the motifs are in boxes colored as in (A) to (E). Abbreviations: AAC, ADP/ATP carrier; AGC, aspartate glutamate carrier; APC, ATP-Mg/Pi carrier; CAC, carnitine-acylcarnitine carrier; CIC, citrate (tricarboxylate) carrier; OGC, oxoglutarate carrier; PIC, phosphate carrier; TPC, thiamine pyrophosphate carrier; UCP1, uncoupling protein.

A striking feature of the m-state structure is the close packing of the transmembrane helices towards the intermembrane side of the mitochondrial inner membrane (Figures 2 and 5), both within domains (intradomain) and between domains (interdomain). This positioning of helices allows the cytoplasmic network to form in the m-state and blocks access to the substrate-binding site from the intermembrane space [5,54]. For the SLC25 family, two conserved sequence elements permit the close approach of the helices in the m-state: a πGπxπG motif on the odd-numbered helices and a πxxxπ motif on the even-numbered helices (Figure 5), where *π* is the one letter code for amino acids with small side chains. At the interhelical interface of each domain, the glycines and middle π residue of the πGπxπG motif on the odd-numbered helix lie opposite the πxxxπ motif on the facing even-numbered helix (Figure 5B–E) [54]. The flanking π residues of the πGπxπG motif face the interdomain interfaces (Figure 5B). The small size of the side chains in both motifs permits the close packing of helices. The residues of these motifs lie far apart in the c-state structures (Figure 5A).

Originally, the πGπxπG motif was called the GxxxG motif [5,54], but comparison of a wide range of SLC25 sequences suggests that the motif can be expanded (Figure 5F). Mutation of these residues in the oxoglutarate carrier significantly reduces or abolishes transport activity [67]. It should be noted that occasionally amino acids with bulkier side chains are found in the π positions of both motifs (Figure 5F, G). In these instances, it appears that they can be accommodated by orienting the side chain away from the interface, so that it points either inside the protein cavity, or towards the lipid bilayer (Figure 5E).

The conservation of these features indicates that all mitochondrial carriers share a mechanism that permits the close approach of the transmembrane helices on the intermembrane side, which is critical for the formation of the cytoplasmic network.

## Energetics of Transport

By analogy with enzyme kinetics, Klingenberg introduced the concept of ‘induced transition fit’ for carrier catalysis [72]. He proposed a transition state between c- and m-states, in which the substrate-binding site has the optimal fit to the substrate. Substrates initially bind to the c- or m-state weakly, but induce conformational changes that lead to tighter binding and the formation of the transition state. The carrier-substrate binding energy lowers the activation barrier for formation of the transition state, catalyzing transport.

**Figure 6:**
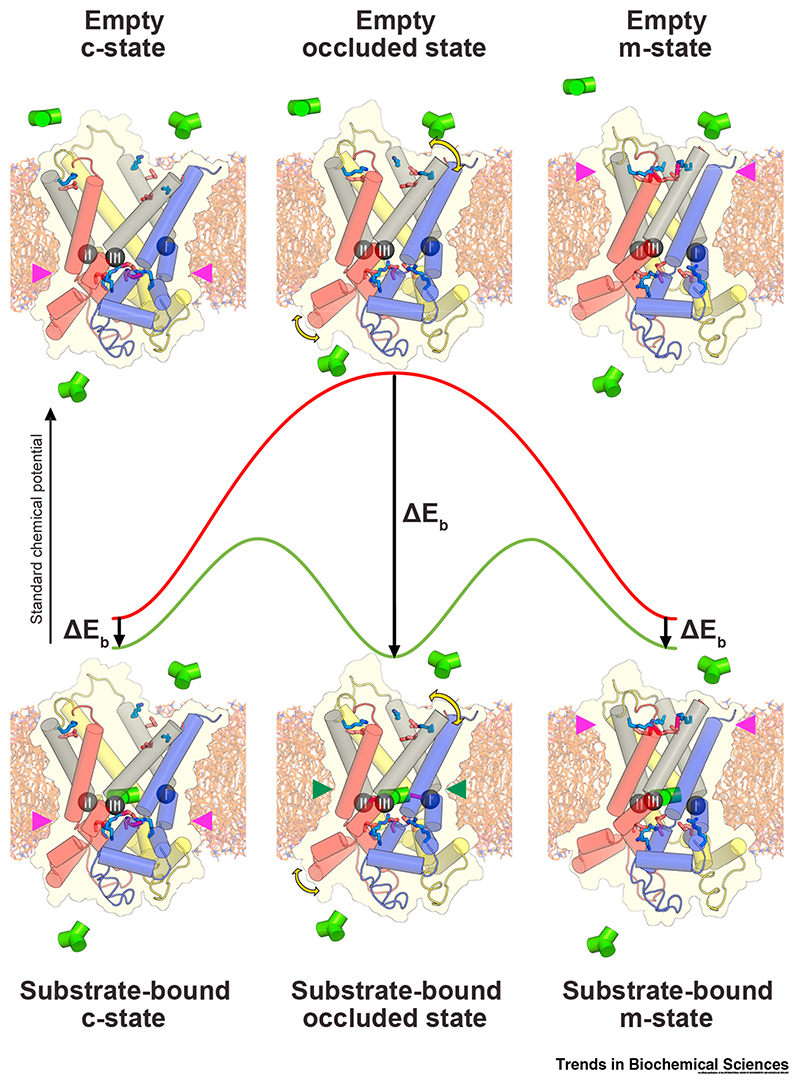
Structures and Energetics of Intermediates in the Mitochondrial Carrier Transport Cycle. The structures of the cytoplasmic (c-), matrix (m-) and occluded states are based upon models of the uninhibited mitochondrial ADP/ATP carrier [54] and are shown with cylindrical helices, colored blue, yellow, and red for the core elements of domains 1, 2, and 3, respectively, with the gate elements in grey. The cytoplasmic and matrix gate residues are shown as sticks, with interactions shows as magenta dots when the gates are closed (magenta arrowheads). The contact points of the substrate-binding site (green arrowheads) are shown as black spheres with Roman numerals. Substrate (green trigonal object) binding to the carrier reduces the energy state of the intermediates. This is most pronounced for the occluded state, the substrate-binding site of which optimally interact with the substrate, lowering the energy barrier for transport. Figure adapted, with permission, from [71].

Mitochondrial carriers appear to have no conserved interactions between their dynamic elements, other than the salt-bridge networks [49]. Substrate binding provides the energy input for disruption of the networks. A Markov model of the ADP/ATP carrier has been developed, which treats it as a nanomachine that can move stochastically between states under the influence of thermal energy, with the free energy profile being determined by the strength of substrate binding and the two salt-bridge networks [71]. The analysis showed that optimal transport rates are observed only when the interaction energies in substrate binding and the matrix and cytoplasmic salt-bridge network are approximately equal (Figure 6) [71]. The transport model predicted that the carrier has an intermediate substrate-bound **occluded state**, which is supported experimentally by the observation that it can be stabilized [57]. The model also anticipated that there had to be additional polar interactions in the cytoplasmic network, which subsequently turned out to be the tyrosine braces [54].

The concept that the substrate-bound c-, occluded, and m-states are on similar energy levels is also supported by the available structural information. The matrix salt-bridge network of the human ADP/ATP carrier has three ionic interactions and one glutamine brace, whereas the cytoplasmic salt-bridge network is marginally stronger, with three ionic interactions and two tyrosine braces (Figure 4). Our current understanding of substrate binding suggests that there are three ionic interactions between the phosphate groups of the substrates and the positively charged residues of the binding site, an aromatic stacking interaction (equivalent to a hydrogen bond), and one hydrogen bond (Figure 3C). The marginal stronger cytoplasmic network could be disrupted by the binding of ATP, which has a slightly higher binding energy than ADP [57] and is the principle physiological substrate during the m- to c-state transition.

In principle, the proposed transport mechanism is fully reversible and directionality is imposed by chemical gradients of the substrates and the proton-motive force. Extending the above analysis, there are at least three important principles relating substrate-binding to the conformational changes required for transport in other mitochondrial carriers. First, energy released by substrate binding drives the conformational changes between c- and m-states and needs at least to match the interaction energy of the disrupted network. Second, **proton coupling** is an additional consideration, since binding of protons to residues in the binding site will also release energy that can contribute to network disruption, enabling substrates that might form few interactions to disrupt a relatively strong network (e.g., PIC, AGC, GC, and CIC) (Figure 4) [17]. Furthermore, proton coupling will force the symport of a cosubstrate, even against its own chemical gradient. Third, a relatively weak network has a higher probability of disrupting spontaneously, potentially enabling a transition between c- and m-states to occur without substrate, allowing uniport (e.g., TPC) [51]. For most carriers, these considerations reflect their transport properties fairly well, but given the strength of the cytoplasmic network, UCP1 and PIC would be predicted to have counter-substrates.

## Peculiar Family Members

Four SLC25 members are peculiar, three of which have been reported to localize to the mitochondrial outer, rather than inner, membrane. MTCH1 (SLC25A49) [73] and MTCH2 (SLC25A50) [74] have been implicated in apoptosis and SLC25A46 in mitochondrial fission [75]. Sequence analysis indicates that all three proteins have a similar topology to other mitochondrial carriers. In all three, the prolines of the signature motifs are well conserved, but the charged residues that form the matrix and cytoplasmic salt-bridge networks are not. In addition, MTCH2 appears to have a truncated H1 and a highly divergent sequence for H6, suggesting that the local structure of this helix may be different [76], which might also hold for MTCH1. SLC25A46 appears to have a truncated matrix helix h34. The other unusual family member is PMP34 (SLC25A17), which localizes to peroxisomes [77] and, unusually, has a weaker matrix salt-bridge network compared with the cytoplasmic network.

## Concluding Remarks

The structure of a mitochondrial ADP/ATP carrier in the m-state, combined with earlier structures of the c-state, have provided the first structural mechanism for transport by the SLC25 family. Key elements, such as the salt-bridge networks, gates, substrate-binding site, and πGπxπG and πxxxπ motifs play important roles in the transport mechanism. Since these sequence features are highly conserved among SLC25 family members, it is likely that they share a similar transport mechanism. Despite these advances, there are many questions that remain to be addressed for this fascinating protein family (see Outstanding Questions). In particular, research needs to address how mutations can cause dysfunctional carriers, resulting in a range of neuromuscular, metabolic, and developmental disorders.

## Highlights

Mitochondrial carriers, belonging to the SLC25 family of transport proteins, are essential for the functioning of eukaryotes because they transport nutrients across the mitochondrial inner membrane for energy conversion and maintenance of the cell.

The recently determined structure of a mitochondrial ADP/ATP carrier in the inhibited matrix-state, combined with earlier structures in the inhibited cytoplasmic state, provides the first description of the transport mechanism for an SLC25 protein.

Sequence elements, conserved across the mitochondrial carrier family, play important roles in the mechanism, pointing to a common transport mechanism across SLC25 proteins.

Mitochondrial carriers are the most dynamic transport proteins yet characterized, undergoing large conformational changes, allowing them to transport some of the largest biochemical compounds without significant proton leak.

## Glossary

### Abbreviations

Alternating access: a transport mechanism used by most secondary active transporters in which the substrate-binding site is exposed to one or other side of the membrane in two different conformational states, via the coupled opening and closing of gates.
Antiporter: secondary active transport protein that exchanges molecules or ions across a biological membrane; usually the electrochemical gradient of one substrate drives the transport of the other.
Contact point: a residue, or set of residues, on a helix of an SLC25 carrier that interacts with the substrate in the binding site.
Cytoplasmic salt-bridge network: the second and fifth residues of the conserved [YF][DE]xx[KR] motifs, frequently charged, which can form interactions between the even-numbered α-helices of SLC25 proteins in the matrixstate.
Cytoplasmic state (c-state): a conformational state of an SLC25 carrier in which its central substrate-binding site is accessible to the mitochondrial intermembrane space, which is confluent with the cytosol.
EF-hand: a helix-loop-helix structural motif found in proteins, capable of binding calcium ions, which can lead to a conformational change.
Gate: part of a transport protein that, in one conformational state, blocks access of substrate from one side of the membrane to its binding site.
Matrix salt-bridge network: the third and sixth conserved residues of the Px[DE]xx[KR] signature motifs, frequently charged, which can form interactions between the odd-numbered α-helices of SLC25 proteins in the cytoplasmic state.
Matrix state (m-state): a conformational state of an SLC25 carrier in which its central substrate-binding site is accessible to the mitochondrial matrix.
Occluded state: an intermediate state in the transport cycle, in which both gates are closed, preventing uncontrolled movement of molecules and ions across the membrane.
Proton coupling: a special case of symport, in which the transport of a substrate is coupled to the transport of one or more protons.
Q brace: amino acid residues lying on the matrix side of the matrix salt-bridge networks, frequently glutamines; they can form hydrogen bonds that help to stabilize the matrix salt-bridge network.
Signature motif: highly conserved amino acid sequence Px[DE]xx [KR]; found on the odd-numbered α-helices of all three domains of SLC25 members, the prolines lie at the kinks of the helices (P-kink) and the charged residues form the matrix salt-bridge network.
Symporter: secondary active transport protein that co-transports two or more molecules or ions in the same direction across a biological membrane, at least one of which is moving thermodynamically down its electrochemical gradient
Uniporter: transport protein that transports a single type of molecule or ion across a biological membrane down its electro-chemical gradient.
Y brace: amino acid residues lying on the matrix side of the cytoplasmic salt-bridge networks; frequently tyrosines, they can form hydrogen bonds that help to stabilize the cytoplasmic salt-bridge network.

## Outstanding Questions

Many members of the mitochondrial carrier family in humans have not been functionally characterized; what are their substrates? This information is essential to understand their role in mitochondrial and cellular physiology and disease.

Many SLC25 members have multiple isoforms. What is their physiological role? When and where are they expressed on the protein level? What is the implication for mitochondrial diseases?

What is the transport mode of the different SLC members? The physiological role has been established for most members, but for many it has not been definitively established whether they operate by antiport, symport, or uniport mechanisms.

Are SLC25 proteins distributed evenly over the mitochondrial inner membrane, or are they localized, for example, to the cristae?

What is the kinetic mechanism of mitochondrial carriers? All available structural information points to a ping-pong exchange mechanism, but kinetic analyses of transport have suggested a simultaneous exchange mechanism.

How do the structures of substrate-free states differ from the currently available inhibited states? Structural comparisons have indicated that the inhibited carriers are in an abortive state, showing structural perturbations, but what are they?

Are the binding poses of ADP and ATP in the substrate-bound states the same? The current structural information suggest that the same site is involved but are the same residues involved in the binding of ADP and ATP?

How is substrate binding coupled to the conformational changes in the matrix and cytoplasmic state transitions?

How is substrate selectivity achieved? For instance, it is not fully understood how the ADP/ATP carrier binds ADP and ATP, but not the chemically related GDP and GTP.

Is it possible to trap and structurally characterize the occluded state of a mitochondrial carrier? Is the occluded state a transition or intermediate state?

How are the regulatory domains of AGC and APC positioned with respect to the carrier domains? How do calcium-dependent conformational changes in the AGC or APC regulatory domains regulate the transport activity of the carrier domains?

How do mutations lead to dysfunctional carriers and how does this lead to pathophysiological processes in neuromuscular, metabolic, and developmental disorders?
